# Beyond Glucose and Lipids: The Pivotal Role of Amino Acid Reprogramming in Shaping the Tumor Microenvironment and Guiding the Development of Novel Therapeutics

**DOI:** 10.7150/ijms.124859

**Published:** 2026-08-11

**Authors:** Xuanhao Wu, Xiaodan Mao

**Affiliations:** 1 Fujian Clinical Research Center for Gynecological Oncology, Fujian Maternity and Child Health Hospital, Fuzhou 350001, Fujian, China; 2 College of Clinical Medicine for Obstetrics & Gynecology and Pediatrics, Fujian Medical University, Fuzhou 350001, Fujian, China

**Keywords:** Metabolic reprogramming, Amino acid metabolism, Tumor microenvironment, Targeted therapy, Therapeutic strategy

## Abstract

Metabolic reprogramming is a hallmark of tumor initiation and progression. Previous studies have focused mainly on glucose and lipid metabolism, with emphasis on the Warburg effect and *de novo* lipogenesis. Based on the shift in research paradigms, this review focuses on amino acid metabolism, which has long been overlooked, and systematically elaborates how tumors hijack amino acid pathways to construct an immunosuppressive and protumor microenvironment. We confirm that amino acid metabolism acts as a core driver of tumor proliferation, immune evasion and therapeutic resistance. This paper further discusses the intricate regulatory crosstalk between amino acid metabolism and convent ional antitumor therapies, clarifying the potential of metabolic-targeted strategies in overcoming drug resistance and improving clinical efficacy. Deviating from the traditional perception that amino acids merely serve as auxiliary substances for glucose and lipid metabolism, we summarize their vital roles in remodeling the immunosuppressive tumor microenvironment and provide theoretical support for the clinical integration of precise metabolic targeted therapy and existing antitumor regimens combined with cutting-edge technologies.

## Introduction

The metabolic reprogramming of tumors refers to the adaptive process through which cancer cells globally reshape their metabolic pathways. While classically characterized by a shift from mitochondrial oxidative phosphorylation to aerobic glycolysis (the Warburg effect) to reduce oxygen dependency and enable survival in nutrient-deprived and hypoxic microenvironments, this phenomenon extends far beyond energy production. Crucially, it serves to divert metabolic intermediates into biosynthetic pathways, generating the nucleotides, amino acids, and lipids essential for rapid cell proliferation. In addition to that of glucose, tumors frequently rewire the metabolism of amino acids and lipids, establishing a highly flexible metabolic network that supports their uncontrolled growth and resistance to therapeutic stress 1. Numerous studies have demonstrated that glycolytic acidification by cancer cells not only promotes the activation of matrix metalloproteinases (MMPs) but also compromises immune function. Specifically, this acidic microenvironment suppresses CD8⁺ T-cell activity and induces M2 macrophage polarization, thereby driving invasive metastasis in various malignancies, including breast, liver, and prostate cancers 2, 3. However, the metabolic rewiring of the TME affects more than just glycolysis. Complementing the effects of acidification, the dysregulation of amino acid metabolism serves as a pivotal mechanism through which tumors orchestrate their surrounding niche.

Anticancer agents targeting amino acid metabolism are increasingly entering the clinical arena, offering strategic interventions to circumvent the resistance mechanisms of conventional therapies. Notable examples include the glutaminase inhibitor CB-839, which impairs tumor bioenergetics by blocking glutaminase (GLS), and the arginine-depleting enzyme ADI-PEG20, which leverages tumor arginine auxotrophy to trigger metabolic catastrophe.

Thus, amino acid metabolism is an indispensable process in tumorigenesis and tumor progression 4. Going beyond the conventional focus on glucose and lipid metabolism, this review positions amino acid metabolism as a central orchestrator of the immunosuppressive tumor microenvironment, immune evasion, and multidrug resistance. Furthermore, we outline a roadmap for the development of precision and dynamic metabolic targeting strategies harnessing cutting-edge technologies such as spatial metabolomics, nanodelivery systems, and artificial intelligence.

## 1. Role of Amino Acid Metabolism in Tumorigenesis and Progression

In addition to functioning as mere metabolic substrates for proliferation, amino acids act as pivotal signaling nodes orchestrating malignant phenotypes. Tumor cells develop auxotrophy via metabolic reprogramming, a process that is intrinsically linked to immune evasion. Furthermore, this metabolic reliance dictates symbiotic tumor-microenvironment crosstalk: by coopting amino acid resources, cancer cells actively modulate immune and stromal compartments to construct permissive, tumor-promoting metabolic niches. While essential for general physiology, specific amino acids—particularly glutamine and arginine—emerge as critical orchestrators within the tumor immune microenvironment, driving immunosuppression, malignant growth, and therapeutic resistance. These metabolites underpin a complex network of processes ranging from biosynthesis and catabolism to transport and signal transduction. In this review, we specifically delineate the mechanisms by which these key amino acids shape the metabolic landscape of cancer.

### 1.1. Glutamine (Gln) and Its Metabolites

**Figure 1:**
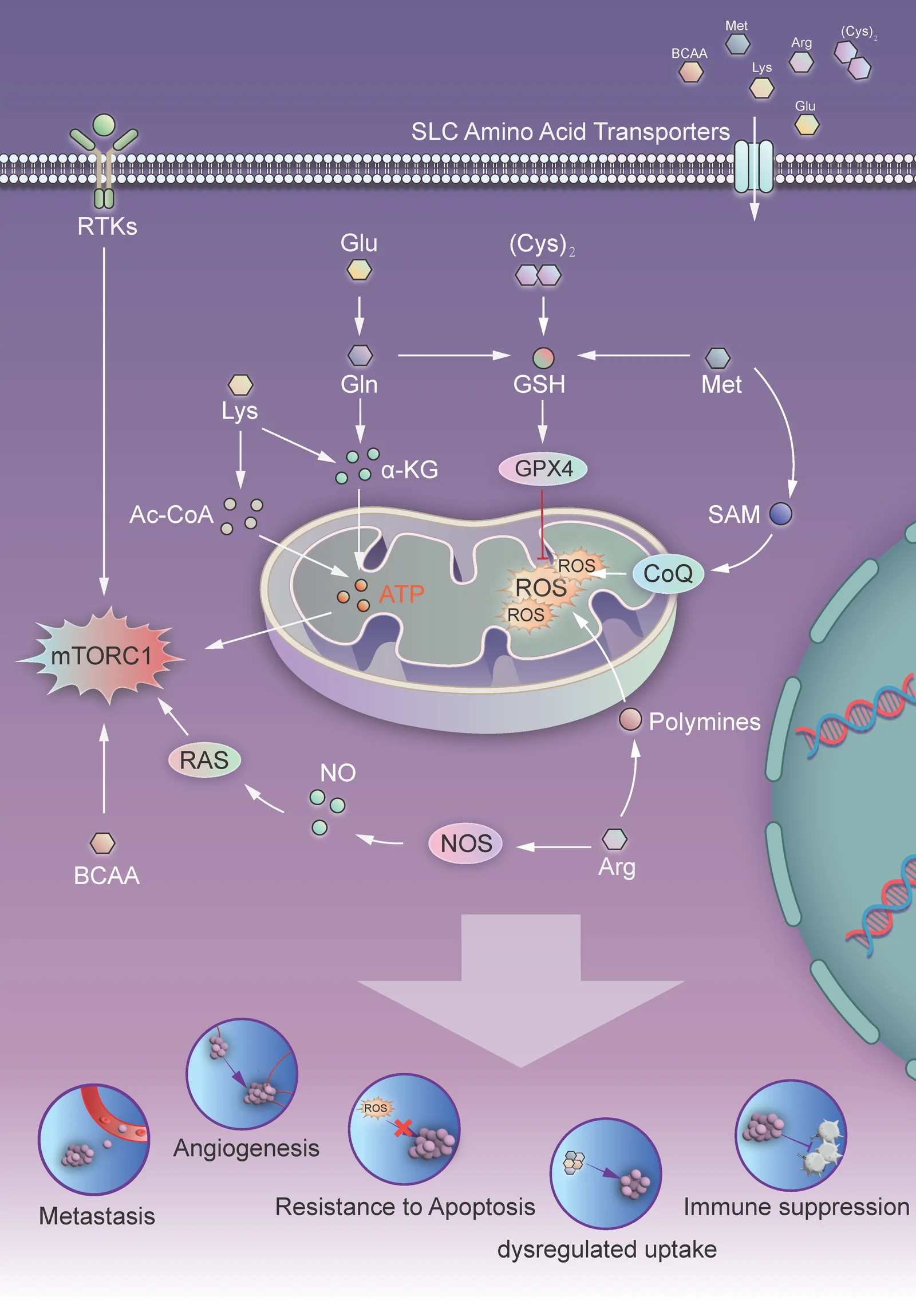
** Amino Acid-Driven Oncogenesis: Model of Metabolic Reprogramming and Progression Pathways.** The white arrows indicate activation/stimulation, whereas the red T-bar arrows (⊥) represent inhibition/suppression. Glu, Glutamate; Gln, Glutamine; (Cys)₂, Cystine; Lys, Lysine; Met, Methionine; Arg, Arginine; BCAA, Branched-chain amino acid; SAM, S-adenosyl methionine; GSH, Glutathione; α-KG, Alpha-ketoglutarate; Ac-CoA, Acetyl-coenzyme A; CoQ, Coenzyme Q; ROS, Reactive oxygen species; NOS, Nitric oxide synthase; RAS, RAS pathway; RTK, Receptor tyrosine kinase; mTORC1, Mechanistic target of rapamycin complex 1; GPX4, Glutathione peroxidase 4.

Although glutamine (Gln) is classified as a nonessential amino acid because of its capacity for *de novo* synthesis, it becomes conditionally essential during periods of severe physiological stress, including major surgery, bone marrow transplantation, and intensive chemoradiotherapy. The metabolic flux between Gln and glutamate (Glu) is governed by glutamine synthetase (GS) and glutaminase (GLS) (Fig. 1). This dynamic equilibrium not only links the two amino acids but also serves as a critical metabolic node in tumor biology 5. Tumor cells hijack glutamine (Gln) transport mechanisms to satisfy their heightened demand for carbon and nitrogen skeletons. This metabolic reprogramming supports rapid proliferation and malignant progression by providing key building blocks for biosynthesis, including ribose-5-phosphate, citrate, and glycerol-3-phosphate 6.

In 1955, Eagle H. first demonstrated the high-glutamine (Gln) dependence of tumor cells *in vitro*. Clinical studies have revealed that mesenchymal tumors with high metastatic potential—including pancreatic ductal adenocarcinoma (PDAC) and triple-negative breast cancer (TNBC)—are particularly susceptible to RAS oncogene-induced ferroptosis 6. In this pathway, glutathione peroxidase 4 (GPX4) utilizes glutathione (GSH) to suppress ferroptosis. However, the biosynthesis of GSH is strictly dependent on the activation of the cystine-glutamate antiporter SLC7A11, which is itself regulated by the availability of glutamine (Gln) 7**.** Current research has established that SLC7A11 overexpression serves as a pivotal determinant of ferroptosis resistance in tumor cells 8 (Fig. 1). Functioning as a 1:1 antiporter, SLC7A11 exchanges extracellular cystine for intracellular glutamate (Glu), causing a marked efflux of Glu. The resulting accumulation of extracellular Glu exerts negative feedback inhibition of SLC7A11, which compromises intracellular GSH synthesis and GPX4 activity. This metabolic dysregulation culminates in lipid peroxidation and ferroptotic cell death, effectively abrogating tumor progression 9, 10.

Since the original study by Reitzer L. J. et al. in 1979, it has been established that tumor cells favor glutamine (Gln) over glucose for redox maintenance, even when glucose is abundant. In endometrial cancer, estrogen drives this metabolic reprogramming by increasing the activity of glutamine synthetase (GS) to ensure Gln availability. Mitochondrial Gln metabolism generates malate via the TCA cycle, which is subsequently exported to the cytoplasm. Malic enzyme 1 (ME1) converts malate into NADPH, fueling the glutathione (GSH) and thioredoxin (TRX) systems. Consequently, this metabolic axis confers robust resistance against ferroptosis 11, 12. In ovarian cancer (OC), Gln increases glycolysis via the mTOR pathway, which supplies additional energy to support tumor growth. Gln depletion suppresses tumor progression. Conversely, Gln deficiency triggers a compensatory survival response. It upregulates c-Myc expression, which drives the transcription of GOT1 and NRF2 to sustain GSH synthesis. Moreover, it activates NRF2 to induce G6PD expression. This dual regulatory axis orchestrates metabolic adaptation, allowing tumors to withstand metabolic stress within the TME 13, 14.

Extensive research has demonstrated that Gln metabolism plays a pivotal role in tumor immune evasion. Excessive Gln uptake by tumor cells not only restricts the availability of Gln to immune cells, thereby impairing antitumor immunity, but also triggers the upregulation of programmed death-ligand 1 (PD-L1). This metabolic reprogramming mediates T-cell apoptosis and ultimately promotes immune escape 14. Furthermore, tumor-derived glutamate engages surface receptors on neutrophils, triggering the upregulation of pSTAT3, ARF4, and RAB10 expression. This signaling cascade orchestrates a functional shift in neutrophils, polarizing them from a proinflammatory state to an immunosuppressive state. Consequently, this phenotypic reprogramming severely reduces the cytotoxic effects of neutrophils against tumor cells 15.

In summary, the glutamine-SLC7A11 metabolic axis orchestrates a broad spectrum of tumor-promoting processes. It not only governs glutathione synthesis, ferroptosis resistance, and redox-glycolytic homeostasis but also actively mediates immune evasion by reprogramming neutrophils toward an immunosuppressive, protumorigenic phenotype.

### 1.2. Arginine (Arg) and Its Metabolites

The biosynthesis of arginine (Arg) is canonically catalyzed sequentially by argininosuccinate synthase (ASS1) and argininosuccinate lyase (ASL). In the context of the tumor microenvironment (TME), Arg metabolism bifurcates into two critical axes—the urea cycle and the nitric oxide synthase (NOS) pathway—which collectively orchestrate tumorigenesis and malignant progression.

Seminal studies from 1975 revealed a critical metabolic liability in melanoma and hepatocellular carcinoma (HCC): auxotrophy for exogenous arginine (Arg) driven by deficiency of argininosuccinate synthase (ASS1) or ornithine transcarbamylase (OTC). This dysregulation of the urea cycle is a key driver of tumorigenesis. Additionally, Arginase-1/2 (ARG1/2) catalyzes the hydrolysis of Arg into urea and ornithine (Orn). Beyond serving as a precursor for polyamine synthesis, Orn actively skews macrophages toward an M2-like phenotype. This process promotes an immunosuppressive microenvironment, thereby creating a feedback loop that further accelerates malignant progression 16, 17.

Furthermore, the aberrant accumulation of polyamines undermines the cytotoxic effects of NO in macrophages on tumor cells. It achieves this by competing for L-arginine, skewing macrophages toward an M2 phenotype, and altering the oxidative landscape. These alterations establish a metabolic framework that rationalizes the concentration-dependent dual functionality of NO 18, 19. Aberrant polyamine accumulation plays paradoxical roles in the tumor microenvironment. On the one hand, it triggers hydrogen peroxide-mediated ferroptosis, whereby the release of polyamines from dying cells propagates cell death and exerts tumor-suppressive effects. Conversely, excessive polyamines promote tumorigenesis and tumor progression by stimulating angiogenesis and cellular proliferation while simultaneously amplifying the immunosuppressive activity of myeloid-derived suppressor cells (MDSCs) 20 (Fig. 1). It is currently believed that nitric oxide (NO) has a concentration-dependent “double-edged sword” effect *in vivo*. At low concentrations, it drives tumor progression through the VEGF, ERK, and mTOR signaling pathways; conversely, at high concentrations, it mediates antitumor effects by inducing apoptosis and inhibiting respiratory chain complexes 21. Recent studies indicate that Arg metabolism is significantly reprogrammed within the TME. The resulting fluctuations in Arg levels perturb the TCA cycle and fatty acid metabolism, consequently orchestrating tumor initiation and progression 22, 23. However, significant hurdles persist in elucidating the mechanisms of Arg-mediated NO regulation in the TME, which are governed by metabolic heterogeneity across tumor subtypes and intricate immunomodulatory networks 24.

Tumor cells competitively consume arginine within the tumor microenvironment to limit the bioavailability of arginine to immune cells. By regulating nitric oxide production, arginine exerts concentration-dependent dual protumor and antitumor effects. It also modulates the TCA cycle and fatty acid metabolism and engages in crosstalk with polyamine metabolism, thereby regulating macrophage M2 polarization, ferroptosis, angiogenesis, myeloid-derived suppressor cell (MDSC) function and tumor immune homeostasis.

### 1.3. Tyrosine (Tyr) Metabolism and Tyrosine Kinase Signaling

Tyrosine (Tyr) is a nonessential amino acid obtained from dietary protein or synthesized from phenylalanine by phenylalanine hydroxylase. In human metabolism, tyrosine serves as a precursor for several critical molecules. The primary metabolic pathways involved include its conversion to catecholamines (dopamine, norepinephrine, and epinephrine) in the brain and adrenal glands, the synthesis of thyroid hormones (T3 and T4) in the thyroid gland, and the production of melanin for pigmentation. Alternatively, tyrosine is degraded via tyrosine aminotransferase to p-hydroxyphenylpyruvate and ultimately to fumarate and acetoacetate, which enter the citric acid cycle for energy production or are excreted in the urine. Tyrosine serves as an essential substrate for protein tyrosine kinase-mediated phosphorylation and the initiation of T-cell activation signaling pathways. An insufficient tyrosine supply directly suppresses the activation of tyrosine kinase-driven signaling cascades in immune cells, hinders the proliferation and differentiation of T cells as well as the secretion of effector molecules such as interferons and perforin, impairs the antitumor cytotoxic function of macrophages, induces metabolic stress and functional exhaustion in immune cells, and ultimately mediates tumor immune escape through nutrient deprivation.

Protein tyrosine kinases (PTKs) play pivotal roles in normal cellular physiology by orchestrating essential functions such as growth factor signaling, cell proliferation, and differentiation. Alterations in the abundance of intracellular tyrosine can indirectly affect the level of protein tyrosine phosphorylation, thereby regulating the activity of tyrosine kinase pathways 25 (Fig. 1). Seminal research from 2000 underscores the pivotal role of vascular endothelial growth factor receptor (VEGFR) signaling in tumor angiogenesis, identifying the VEGF/Flk-1/KDR axis as a critical endothelial-specific regulator of neovascularization. Beyond angiogenesis, VEGFR signaling is indispensable for hematopoiesis, MMP activation, and the recruitment of monocytes and other immune cells into the TME. Mechanistically, the binding of VEGF to VEGFR-2 activates the PI3K pathway, thereby orchestrating endothelial cell survival, proliferation, and angiogenesis to promote tumor progression. Furthermore, aberrant VEGF-C expression and downstream VEGF-C/VEGFR signaling are significantly correlated with poor prognosis across a spectrum of malignancies 26. Epidermal growth factor receptor (EGFR) is among the most extensively studied receptor tyrosine kinases. Its phosphorylation governs fundamental cellular processes, including proliferation, survival, migration, and angiogenesis. Across a spectrum of malignancies—including breast, bladder, lung, ovarian, pancreatic, and prostate cancers—aberrant mutations within the EGFR kinase domain lead to constitutive receptor activation. This persistent signaling acts as a potent driver of oncogenesis and actively shapes the TME 27. Elevated EGFR signaling upregulates PD-L1 protein expression in non-small cell lung cancer (NSCLC) cells, thereby suppressing antitumor immunity 28. Furthermore, the PTK domain of human epidermal growth factor receptor 2 (HER2) is a frequent site of oncogenic mutations. In addition to mutations, HER2 amplification and overexpression are characteristic features of a diverse spectrum of malignancies, including breast cancer (BC), gastric cancer (GC), non-small cell lung cancer (NSCLC), cholangiocarcinoma, bladder cancer (BCa), and colorectal cancer (CRC) 29. However, the mechanisms underlying these genetic changes remain unclear 30.

In addition to direct oncogenic signaling, PTK activity promotes tumor progression through the regulation of immune checkpoints. Emerging evidence indicates that Axl, a receptor tyrosine kinase expressed in multiple cancer types, is involved in the Gas6/Axl signaling pathway and is strongly associated with therapeutic resistance and poor clinical outcomes 31. Furthermore, a 2024 study revealed that focal adhesion kinase (FAK) drives tumor immune evasion and metastasis by regulating immune cell functions within the TME, including by suppressing T-cell activity and promoting the differentiation of MDSCs 32. The activation of receptor PTKs, such as EGFR and MET, has been shown to trigger PD-L1 (CD274) transcription, which is largely mediated by signaling cascades such as the IL-6/JAK/STAT3 axis. Clinically, PD-L1 upregulation frequently occurs in patients receiving EGFR-TKI therapy; this overexpression is correlated with acquired resistance and hyperprogressive disease following treatment with PD-1/PD-L1 checkpoint inhibitors 33.

Protein tyrosine kinases (PTKs) are aberrantly activated in tumors, driving cancer initiation and progression by regulating key pathways such as the Ras, mTOR, PI3K, and IL-6/JAK/STAT3 pathways. Furthermore, they modulate tumor angiogenesis (via VEGFR and EGFR), mediate oncogenic signaling (through EGFR, HER2, Axl, and FAK), and promote tumor immune evasion by regulating immune checkpoints (e.g., PD-L1) and immune cell functions, including T-cell suppression and MDSC differentiation.

### 1.4. Methionine (Met) and Its Metabolites

Methionine (Met), an essential sulfur-containing amino acid, primarily participates in the methionine cycle. Through transmethylation, Met is converted to S-adenosylmethionine (SAM), which serves as the primary methyl donor before being metabolized to S-adenosylhomocysteine (SAH). SAH is subsequently hydrolyzed to homocysteine (Hcy) and finally remethylated to regenerate Met in a folate- and vitamin B12-dependent manner. This fundamental metabolic pathway was first comprehensively characterized by Finkelstein in 1990 34. While Met promotes tumor progression by facilitating GSH and polyamine synthesis and serving as a methyl donor, it also modulates multiple pathways to suppress tumor cell proliferation or induce ferroptosis, playing paradoxical roles in tumor biology.

The metabolic dependency of tumors on exogenous methionine (Met) was first documented by Sugimura in 1959. Subsequent research has demonstrated that Met deprivation destabilizes histone methylation states, particularly H3K4me3 and H3K9me3, suggesting that this epigenetic instability may be a critical determinant of tumor Met dependency 35. Subsequent studies of hepatocellular carcinoma (HCC) have revealed that elevated SAM levels are closely correlated with T-cell exhaustion. Pharmacological inhibition of methionine metabolism induces cellular senescence in HCC, whereas the concurrent depletion of SAM increases susceptibility to glycogen synthase kinase 3 (GSK3) inhibition 36, 37. However, methionine (Met) serves as an essential precursor for the synthesis of glutathione (GSH). Prolonged Met deficiency disrupts general protein synthesis, thereby maintaining GSH levels above a lethal threshold. Conversely, excess Met elevates intracellular GSH levels, which confers drug resistance in malignancies such as multiple myeloma and breast cancer 38.

Early 20th century studies in animal models revealed that beyond its direct effects, S-adenosylmethionine (SAMe) downregulates transferrin receptor (TfR) expression, thereby reducing hepatic iron uptake and subsequent ferroptosis. These findings highlight the intimate link between methionine (Met) metabolism and iron homeostasis. Recent research has further demonstrated that the Met-SAM axis actively participates in ferroptosis activation by regulating coenzyme Q (CoQ) biosynthesis. This regulation maintains mitochondrial electron transport chain (ETC) function, promoting reactive oxygen species (ROS) accumulation and indirectly facilitating ferroptosis 39 (Fig. 1). Additionally, the methionine cycle generates substantial amounts of homocysteine (Hcy), a toxic metabolite associated with oxidative stress and apoptosis. Intriguingly, hydrogen sulfide (H₂S) mitigates these deleterious effects. Furthermore, H₂S is frequently upregulated in tumor cells to sustain proliferation, growth, survival, and migration 40.

In a pioneering study in 2020, Bian et al. elucidated the critical crosstalk between methionine (Met) cycling and the immune microenvironment. They revealed that tumors actively sequester environmental Met via SLC43A2, thereby inducing S-adenosylmethionine (SAM) scarcity in T cells. This metabolic constraint disrupts histone methylation patterns (e.g., H3K79me2), which subsequently abrogates T-cell proliferation and effector functions 41. Furthermore, a 2024 study revealed that tumor-derived Met promotes angiogenesis in OC models by modulating the expression of suppressor of cytokine signaling 2 (SOCS2) to induce M2 macrophage polarization 42. Tumors utilize S-adenosylmethionine (SAM), which is generated through methionine metabolism, to drive the m⁶A modification of mRNA, thereby promoting the translation of immune checkpoint genes such as PD-L1 and VISTA. This modification leads to upregulation expression of these immunosuppressive molecules on the tumor cell surface, which directly suppresses the cytotoxic activity of T cells 43, 44.

Met plays a pivotal role in regulating tumor progression and the TME. Its functions include mediating histone methylation, modulating glutathione (GSH) levels and ferroptosis via the Met-SAM axis, impairing T-cell function, promoting M2 macrophage polarization, and driving the m-A modification of immune checkpoint genes such as PD-L1 and VISTA.

### 1.5. Branched Chain Amino Acid (BCAA) Metabolites

Branched-chain amino acids (BCAAs), comprising leucine, isoleucine, and valine, were first systematically characterized by Harper et al. in 1984. The canonical BCAA metabolic pathway involves transamination by branched-chain amino acid transferases (BCATs) to generate branched-chain α-keto acids (BCKAs). This is followed by oxidative decarboxylation via the branched-chain α-keto acid dehydrogenase (BCKDH) complex, yielding TCA cycle intermediates (e.g., acetyl-CoA and succinyl-CoA) for energy production. Accumulating evidence has demonstrated that multiple metabolites within this pathway are involved in tumor initiation and progression 45.

By overexpressing transmembrane transporters such as LAT1, tumor cells actively take up large amounts of BCAAs from the TME. Activated CD8⁺ effector T cells require sufficient leucine to drive clonal expansion and activate the mTORC1 signaling pathway, thereby secreting effector molecules, including interferon-γ, perforin and granzymes. Nevertheless, in cancer cells with high LAT1 expression, most extracellular leucine is depleted, resulting in the functional suppression of neighboring T cells and markedly impairing their antitumor immune responses. Branched-chain amino acid transferase (BCAT) is a pivotal enzyme intimately linked to tumor metabolism. In 2013, seminal studies revealed that BCAT1 is highly expressed in gliomas, where it promotes tumor proliferation by driving the metabolic reprogramming of branched-chain amino acids (BCAAs) 46. BCAT1-regulated branched-chain amino acids (BCAAs) play dual roles as both metabolic substrates and signaling molecules. By upregulating the expression of Ras homolog family member C (RhoC), BCAAs orchestrate fundamental cellular processes, including cell shape modulation, adhesion, division, migration, and vesicular trafficking. These coordinated activities ultimately accelerate tumor progression and are closely correlated with poor clinical outcomes 47. In HCC models, the suppression of BCAA catabolic enzyme activity leads to BCAA accumulation within the TME. The degree of enzymatic inhibition strongly correlates with tumor invasiveness, suggesting that BCAA enrichment confers a selective survival advantage to malignant cells 48.

Under BCAT catalysis, BCAAs are converted into the corresponding BCKAs, which subsequently enter mitochondria and are processed by the BCKDH complex. The activity of BCKDH is negatively regulated by BCKDH kinase (BCKDK). Tumor cells inhibit BCAA catabolism by upregulating the expression of BCKDK, thereby leading to the accumulation of BCAAs within the tumor microenvironment (TME). Under conditions of impaired BCAA catabolism, persistent BCAA accumulation leads to hyperactivation of mTOR. This not only drives tumor progression but also utilizes amino acid metabolic compensation to sustain mTOR activity even when upstream signaling is attenuated 49, 50 (Fig. 1). However, in 2020, BCKDH was found to be upregulated in CRC and to be associated with metastasis and poor prognosis 51, 52. Subsequent studies revealed that BCKDH is upregulated in NSCLC and promotes tumor progression by enhancing glycolysis 53. This implies that BCAA metabolism is distinct across different tumor types. Moreover, BCAAs regulate the expression of the transcription factor c-MYC, which indirectly promotes mRNA translation to increase VEGF expression—a mechanism that has been observed in both breast cancer (BC) and gastric cancer (GC) 54, 55.

Furthermore, tumor cells avidly take up branched-chain amino acids (BCAAs) by highly expressing transporters such as LAT1. BCAAs serve as critical carbon sources for *de novo* lipid synthesis, supporting cancer cell membrane biogenesis, sustaining mTORC1 pathway activation, and ultimately driving cancer cell proliferation 56, 57. In BCa, BCAT2 suppresses the activity of T-cell chemotaxis pathways, thereby inhibiting lymphocyte recruitment and ultimately promoting tumor progression 58. Therefore, the tumor-specific heterogeneity of BCAA metabolism and its interplay with the immune system remain to be further elucidated.

BCAAs regulate tumor progression and the TME via BCAT/BCKDH-mediated metabolism, mTOR activation, c-MYC/VEGF upregulation, and immune modulation (suppressing effector T cells, promoting Tregs, and inhibiting lymphocyte recruitment), with tumor-specific metabolic heterogeneity.

### 1.6. Lysine (Lys) Metabolites

The findings described above imply that BCAA metabolism is distinct across different tumor types. Moreover, BCAAs regulate the expression of the transcription factor c-MYC, which indirectly promotes mRNA translation to increase VEGF expression—a mechanism that has been observed in both breast cancer (BC) and gastric cancer (GC) 59. The metabolic derivatives α-ketoglutarate (α-KG) and acetyl-CoA exhibit dual dependency in tumorigenesis and tumor progression (Fig. 1).

α-KG serves as an essential cofactor for JMJD family histone demethylases. Subsequent studies have revealed that IDH mutations drive the reduction of α-KG to the oncometabolite 2-hydroxyglutarate (2-HG). This metabolite competitively inhibits histone demethylases (KDMs), ultimately promoting leukemogenesis 60. Carey, B. W. et al. demonstrated that α-KG regulates stem cell identity through epigenetic modifications, suggesting its pivotal role in stem cell biology 61. In fact, α-KG not only plays multifaceted roles in cellular energy metabolism and redox homeostasis but also modulates PD-L1 expression and suppresses ferroptosis, thereby promoting immune evasion in tumors 62, 63.

However, studies have revealed that α-KG supplementation can restore differentiation in IDH-mutant tumors, indicating that Lys-mediated tumor regulation via α-KG is also likely governed by another Lys metabolite—acetyl-CoA. Although tumor cells rely primarily on aerobic glycolysis, acetyl-CoA derived from mitochondrial oxidative phosphorylation continues to supply energy and biosynthetic precursors for proliferation. In 2015, research demonstrated that acyl-CoA synthetase short-chain family member 2 (ACSS2)-mediated acetyl-CoA synthesis sustains tumor growth under metabolic stress and that ACSS2 inhibition suppresses cancer cell survival 64. Notably, reduced acetyl-CoA biosynthesis significantly suppresses the proliferation of tumor cells, highlighting its pivotal role in cancer metabolism. In addition, under conditions of glucose deprivation, the accumulation of acetyl-CoA triggers apoptosis through p53 acetylation, revealing its tumor-suppressive potential 65.

Additionally, lactate specifically modifies lysine residues on histones through a process known as lactylation (Kla). This epigenetic modification alters chromatin structure, reprogramming immune cells to silence antitumor genes while activating immunosuppressive pathways. Consequently, this leads to substantial infiltration of myeloid-derived suppressor cells (MDSCs) and regulatory T cells (Tregs), fostering an immune - “cold” tumor microenvironment and contributing to resistance to immunotherapy 66, 67.

Collectively, in addition to a few specific DNA or RNA modifications (JMJD family modifications, m⁶A modification of mRNA, etc.) that affect certain types of tumors, multiple amino acids contribute to the formation and maintenance of the tumor microenvironment through analogous redox reactions, mainly via the mTOR and ferroptosis pathways. Lys regulates tumor progression and the TME via α-KG-dependent epigenetic modifications, 2-HG-mediated oncogenesis, acetyl-CoA-related energy metabolism and apoptosis, and lactylation (Kla)-induced immune suppression, with dual regulatory effects dependent on its metabolites.

In addition, tumor cells and immune cells compete fiercely for limited nutrients within the TME, thereby establishing an immunosuppressive immunometabolic landscape. Compared with T cells, tumor cells consume glucose at a far higher rate. Glucose deprivation hinders the metabolic reprogramming of activated T cells from oxidative phosphorylation to glycolysis, ultimately impairing the effector functions of T cells and inducing T-cell exhaustion. Similarly, competition for amino acids—especially glutamine and tryptophan—further suppresses the immune response. Tumor cells overexpress glutaminase to support anabolic processes, depleting extracellular glutamine and inhibiting T-cell proliferation and cytokine secretion. The massive accumulation of lactate resulting from tumor glycolysis further exacerbates this metabolic imbalance: lactate induces acidification of the microenvironment, inhibits the activity of cytotoxic T cells and natural killer (NK) cells, and simultaneously favors the proliferation of immunosuppressive myeloid cell populations.

## 2. Novel Therapeutic Strategies Targeting Amino Acid Metabolism

Malignant tumors currently account for more than 10 million deaths annually worldwide, with mortality rates demonstrating a persistent increasing trend. The established therapeutic modalities for malignancies include conventional therapies, targeted pharmacological agents, and monoclonal antibodies. Nevertheless, conventional treatments suffer from limited therapeutic precision, inducing nondiscriminatory cytotoxicity against both tumor and normal cells, which elicits substantial adverse effects 68.

The intricate crosstalk between amino acid metabolic reprogramming and the tumor microenvironment not only reveals novel molecular targets but also provides a rational basis for the development of small-molecule inhibitors, metabolic modulators, and combination therapies. While significant progress has been made in elucidating the pathogenic roles of dysregulated amino acid metabolism in various malignancies, the clinical translation of related therapeutic agents has encountered considerable challenges, as evidenced by the failure of agents such as epacadostat in phase III trials. A comprehensive overview of the current status of drug development targeting amino acid metabolism, along with an in-depth analysis of the underlying reasons for therapeutic success or failure and unresolved research gaps, is therefore essential to bridge the gap between mechanistic research and clinical application.

Recent advances in characterizing amino acid dynamics within the TME have led to the development of innovative treatment strategies that leverage these metabolic reprogramming events. In particular, therapeutic interventions targeting dysregulated amino acid metabolic pathways are emerging as pivotal solutions to overcome existing therapeutic gaps. Currently, multiple investigational compounds are undergoing clinical trials, demonstrating transformative potential across antimetabolite therapy, immuno-oncology approaches, molecularly targeted treatments, and advanced nanoscale delivery platforms 69, 70.

### 2.1. Antimetabolite Therapy (AMT)

#### 2.1.1. CB-839

**Figure 2:**
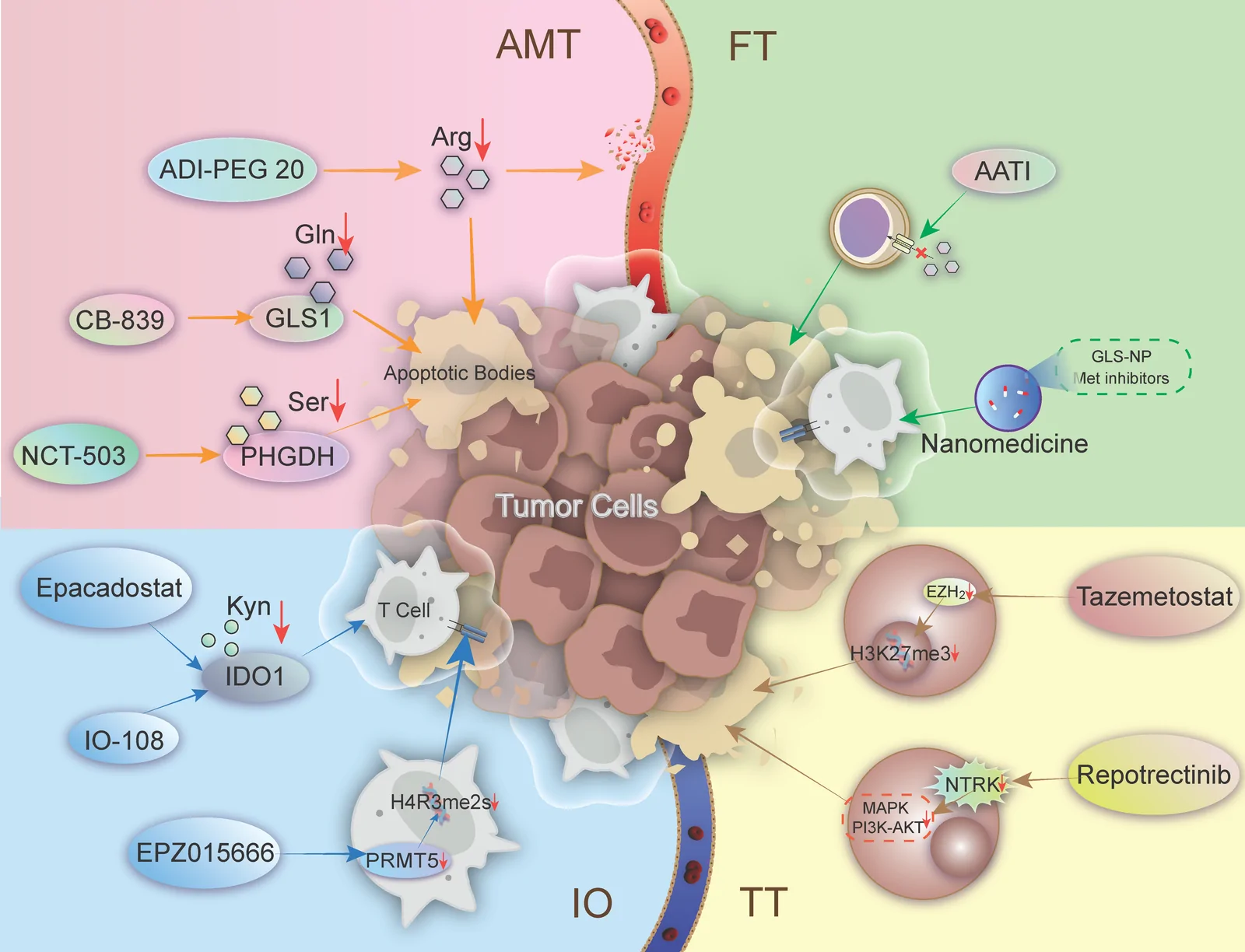
** Target model of drug therapy for tumors.** The red-colored cells represent viable tumor cells, the yellow-colored cells represent apoptotic tumor cells, and the white-colored cells represent T cells. Activated or stimulated T cells are outlined with a white border. The red arrows indicate inhibition of the process, whereas the other colored arrows indicate that the preceding element induces the subsequent element. Ser, Serine; Gln, Glutamine; Arg, Arginine; Kyn, Kynurenine; PHGDH, Phosphoglycerate dehydrogenase; GLS, Glutaminase; IDO, Indoleamine 2,3-dioxygenase; PRMT5, Protein arginine methyltransferase 5; EZH, Enhancer of Zeste homolog; NTRK, Neurotrophic tyrosine receptor kinase.

As an allosteric inhibitor of GLS1, CB-839 exerts therapeutic effects by suppressing the enzymatic activity of GLS1 (Fig. 2). This metabolic intervention leads to significant depletion of intracellular glutamate (Glu) and α-ketoglutarate (α-KG), thereby triggering mitochondrial respiratory chain dysfunction and compromising ATP biosynthesis. Consequently, this results in a dramatic reduction in TCA cycle metabolic flux—often exceeding 50%—in GBM cells 71, 72. Concurrently, blocking glutaminolysis depletes intracellular GSH precursors, leading to ROS accumulation and ferroptosis. Crucially, this metabolic vulnerability also triggers intrinsic apoptotic pathways in CLL models 73.

Current clinical trials of CB-839, primarily conducted in the U.S. and Europe, have demonstrated synergistic antitumor activity when it is combined with cabozantinib in renal cell carcinoma (RCC) (NCT03428217) 74. In a U.S. clinical trial (NCT03163667), the combination of CB-839 and everolimus resulted in a median progression-free survival (PFS) of 3.8 months compared with 1.9 months for the monotherapy arm (HR = 0.64; P = 0.079), a difference that did not reach statistical significance 75. The European data were aligned with the findings from the U.S. study, showing no significant regional differences. Nevertheless, this metabolism-targeting strategy exhibits superior selectivity, reduced off-target effects, and delayed development of resistance—advantages unattainable with conventional therapies 76. Additionally, CB-839 has shown favorable therapeutic outcomes when combined with panitumumab (in CRC), 5-FU (in CRC) and metformin (in OS) (Table 1) 77-79. Moreover, certain epigenetic drugs (e.g., HDAC inhibitors) increase the sensitivity to CB-839, with preclinical models demonstrating a synergy index of 2.4, suggesting a novel direction for future combination therapies 80.

In summary, the success of CB-839 lies in its ability to function not only as a kinase inhibitor but also, more importantly, as a metabolic modulator. It exposes the dependency of tumor cells on amino acids and exploits this reliance as a therapeutic target. In addition to starving cancer cells, CB-839 remodels the tumor microenvironment to increase the oxidative phosphorylation capacity of T cells, thereby activating antitumor immune responses. Rather than being used alone, CB-839 has been strategically paired with a powerful partner—cabozantinib. This “dual-strike” combination has demonstrated significant potential for providing survival benefits in patients with solid tumors such as renal cell carcinoma (RCC), confirming the synergistic advantage of combining metabolic and targeted therapies 81, 82.

#### 2.1.2. ADI-PEG 20

Amino acid-degrading enzymes target tumor metabolic dependencies by depleting essential nutrients in the TME. This metabolic deprivation strategy differs fundamentally from the use of conventional antimetabolites, which typically inhibit enzymes or mimic metabolites. Currently, ADI-PEG 20 stands out as the primary candidate under investigation for this approach.

Owing to the loss of endogenous arginine biosynthesis capacity in ASS1-deficient tumors, malignant cells become obligately dependent on extracellular arginine. This specific metabolic vulnerability is referred to as arginine auxotrophy 83. ADI-PEG 20 (pegylated Arg deiminase) exploits this dependency by catalytically converting circulating Arg into citrulline (Cit) and NH3 to systemically deplete plasma Arg to induce arginine starvation 84, 85 (Fig. 2). Mechanistically, sustained arginine deprivation disrupts the CDK4/cyclin D1 regulatory axis by inactivating the cyclin-dependent kinase complex, culminating in G1/S phase cell cycle arrest in endothelial cells and thereby suppressing neovascularization during tumor progression 86.

While the phase III trials in the United States and Europe (NCT02709512) evaluating ADI-PEG 20 for malignant pleural mesothelioma (MPM) unfortunately failed to meet the primary endpoint—potentially because of resistance from upregulated urea cycle enzymes (e.g., OTC and ARG2) enabling arginine resynthesis—the ATOMIC-Meso Phase III trial yielded contrasting results. It was the first to demonstrate a significant survival benefit (OS = 9.3 vs. 7.7 months; HR = 0.71) when ADI-PEG 20 was combined with first-line chemotherapy (cisplatin plus pemetrexed) in patients with nonepithelioid MPM 85. Regarding combination therapies, clinical trials are currently investigating the efficacy of ADI-PEG 20 in conjunction with pembrolizumab in solid cancers (Table 1) 87.

Notably, arginine-depleting therapies can trigger resistance, as tumors may restore ASS1 expression posttreatment, leading to therapeutic failure 88. ADI-PEG 20 is still in the early phase of clinical trials, and long-term follow-up data are lacking. Research on the mechanisms of drug resistance (e.g., reversal of ASS1 positivity) is insufficient, and the optimal regimen for combination therapy (including drug dosage and administration timing) has not yet been clarified.

#### 2.1.3. NCT-503

The rate-limiting enzyme of the serine synthesis pathway, phosphoglycerate dehydrogenase (PHGDH), is aberrantly overexpressed in many cancers, supporting tumor cell redox homeostasis 89. Preclinical studies have demonstrated that NCT-503 suppresses the *de novo* serine synthesis pathway, impairing glucose-derived citrate biosynthesis, which consequently restricts tumor cell proliferation and survival 90 (Fig. 2). In TNBC models, NCT-503 treatment induces metabolic reprogramming characterized by the downregulation of enzymatic markers (e.g., LDHA and ACLY) while concurrently activating p53-mediated tumor-suppressive pathways to inhibit neoplastic growth 91. Proteomic profiling revealed profound alterations in NCT-503-treated cells across three critical biological axes: metabolic flux regulation, oxidative stress response, and cell cycle checkpoint control 92. Furthermore, a 2021 study in NB models revealed that NCT-503 exhibited off-target effects on glucose metabolism even in PHGDH-knockout cell lines, suggesting its potential additional role through the inhibition of mitochondrial function 90. In 2023, another study reported that in NSCLC, the drug synergistically inhibited glycolysis and serine synthesis pathways when combined with a PKM2 inhibitor (Table 1) 93. Notably, emerging evidence suggests that tumor cells may develop resistance through compensatory pathways or the upregulation of alternative amino acid transporters, representing the primary translational barrier for PHGDH-targeted therapies.

However, subgroup analyses targeting specific patient populations (e.g., individuals with different ages and tumor stages) are lacking for NCT-503, which limits the guidance for precise clinical application. Further in-depth investigations by clinical researchers are therefore warranted.

### 2.2. Immunotherapy (IO)

#### 2.2.1. Epacadostat

Indoleamine 2,3-dioxygenase 1 (IDO1) drives immunosuppressive reprogramming within the TME through the depletion of tryptophan and the concurrent accumulation of immunoregulatory catabolites, such as kynurenine 94. As a heme-dependent competitive IDO1 inhibitor, epacadostat targets the substrate binding pocket in the catalytic domain to restore the tryptophan-kynurenine balance and reverse immunosuppression (Fig. 2). In the pivotal KEYNOTE-434 study conducted in Japan, the safety and tolerability of epacadostat combined with pembrolizumab were assessed in patients with advanced solid tumors. The results indicated that the combination therapy exhibited a favorable safety profile (Table 1) 95.

Although the preclinical results were promising, the pivotal phase III trial (NCT02752074) of epacadostat plus pembrolizumab in melanoma failed to demonstrate significant improvements in the primary endpoints of progression-free survival (PFS) or overall survival (OS). This failure has raised substantial concerns regarding the clinical utility of IDO1 inhibition in cancer immunotherapy. Notably, compared with monotherapy, the combination therapy failed to significantly improve PFS or OS. Several nonexclusive factors may explain this outcome: First, at the dose given, epacadostat (100 mg BID) might not have fully suppressed IDO1 activity. Second, targeting IDO1 alone may be insufficient to reverse the established immunosuppressive tumor milieu. Finally, compensatory upregulation of other tryptophan-catabolizing enzymes (e.g., IDO2 and TDO) could have mitigated the therapeutic effect of epacadostat 96. Therefore, future efforts should focus on three key strategies: first, more aggressive dose escalation or the development of more potent IDO1 inhibitors; second, a paradigm shift from single-target inhibition toward a “broad-spectrum” or “paninhibition” approach that simultaneously blocks multiple nodes (e.g., IDO1, IDO2, TDO) within the complex metabolic network of the tumor microenvironment 97; and third, improving clinical trial design and patient screening to avoid advancing phase III trials solely on the basis of data from single-arm phase II trials. In addition to these factors, issues related to patient stratification and metabolic heterogeneity of the TME remain to be further investigated.

Recent findings have revealed that epacadostat has dual effects: it inhibits IDO1 enzymatic activity but concurrently stabilizes the apo-form of the enzyme, which inadvertently triggers the PI3K/AKT/mTOR pathway and drives tumor proliferation. Given that this protumorigenic effect is most prominent in IDO1-high tumors, precise patient stratification is essential to identify candidates likely to benefit from targeted therapies 98.

#### 2.2.2. IO-108

Leukocyte immunoglobulin-like receptor subfamily B member 2 (LILRB2) is widely expressed across diverse myeloid cells, including monocytes, macrophages, dendritic cells, and neutrophils. Through engagement with multiple ligands (e.g., HLA-G, ANGPTLs, and SEMA4A), LILRB2 suppresses the antigen-presenting function of myeloid cells and modulates tryptophan metabolism-related enzymes (such as IDO), leading to kynurenine accumulation (Fig. 2). IO-108, a fully human monoclonal antibody, exhibits a high affinity for LILRB2 and competitively inhibits its interaction with these ligands. Consequently, the blockade of LILRB2 reverses the immunosuppressive phenotype of myeloid cells, accompanied by a reduction in IDO expression 99. In 2024, the first-in-human study (NCT05054348) evaluated the safety and efficacy of IO-108 as monotherapy and in combination with pembrolizumab (Table 1) 100. Although IO-108 has demonstrated potential in preclinical studies, clinical research remains in the early stages. Moreover, the compensatory upregulation of tryptophan 2,3-dioxygenase (TDO) observed in some patients following IDO/TDO inhibitor administration highlights the necessity of developing dual-target inhibitors of both IDO and TDO.

#### 2.2.3. EPZ015666

PRMT5, a type II arginine methyltransferase, catalyzes the symmetric dimethylation of histone H4R3 (H4R3me2s) and the methylation of various nonhistone substrates, thereby playing pivotal roles in regulating the cell cycle, DNA damage repair, and the maintenance of cancer stem cell properties. The PRMT5 inhibitor EPZ015666 enhances antitumor activity by modulating amino acid metabolism in both T cells and tumor cells and by influencing the expression of key immune checkpoint molecules such as PD-1 and CTLA-4 (Fig. 2) 101. In 2022, a study demonstrated that EPZ015666 overcomes arsenic trioxide (As₂O₃) resistance in acute promyelocytic leukemia (APL) models by destabilizing the PML-RARα oncoprotein, resulting in a 40% higher complete remission (CR) rate than that associated with As₂O₃ monotherapy 102. Preclinical studies have shown that EPZ015666 exerts antitumor effects on retinoblastoma by suppressing the proliferation of retinoblastoma cells 103. Recently, in a breakthrough study, magnetic hydrogel microrobots (MHMs) were developed for the targeted delivery of EPZ015666. This innovative approach significantly increased drug accumulation within tumor tissue and achieved a 3.2-fold increase in the apoptosis rate (p < 0.001) in MTAP-deficient osteosarcoma cells 104. However, high-dose EPZ015666 may inhibit T-cell cytotoxicity, suggesting that how to precisely modulate amino acid metabolism in both T cells and tumor cells has not yet been fully elucidated 105.

### 2.3. Targeted Therapy (TT)

#### 2.3.1. Repotrectinib

The NTRK gene family encodes TRK receptor tyrosine kinases, which play pivotal roles in neural development. Chromosomal rearrangements that involve the fusion of the 3′-end kinase domains of these genes with 5′-end partner genes generate chimeric proteins with constitutive activity, leading to the persistent activation of downstream signaling pathways such as the MAPK, PI3K/AKT, and PLCγ pathways 106. This leads to dysregulated cell proliferation, increased antiapoptotic capacity, and genomic instability 107. Repotrectinib is a novel ROS1/TRK/ALK tyrosine kinase inhibitor (TKI) that blocks signaling pathways triggered by NTRK fusion proteins (Fig. 2).

A 2021 study demonstrated that compared with crizotinib, repotrectinib induces a threefold increase in the apoptosis rate in neuroblastoma (NB), and a 2024 study demonstrated that repotrectinib is tolerable in pancreatic cancer (PC) (with NTRK3 fusion) (Table 1) 108, 109. While repotrectinib significantly delays the onset of drug resistance, long-term monotherapy may inadvertently drive the emergence of unforeseen mutations, underscoring the critical need for future research into combination therapies. Notably, recent studies on tazemetostat have led to a paradigm shift in TRK inhibitor applications—evolving from a broad pancancer approach to a more temporally precise targeting strategy.

Aberrant expression of histone lysine methyltransferases (KMTs) and demethylases (KDMs) is closely involved in tumorigenesis. EZH2, which serves as the catalytic subunit of the polycomb repressive complex 2 (PRC2), catalyzes histone H3 lysine 27 trimethylation (H3K27me3) to mediate transcriptional silencing and drive oncogenic processes. Tazemetostat, a first-in-class EZH2 inhibitor, reduces H3K27me3 levels by competitively binding to the SET domain of EZH2 110 (Fig. 2).

As early as 2020, tazemetostat demonstrated antitumor activity in epithelioid sarcoma and lymphoid malignancies, with tolerability evidenced by a <10% incidence rate of grade 3-4 adverse events 111, 112. Combination therapies integrating tazemetostat with pembrolizumab and topotecan are currently under investigation. Furthermore, recent studies have demonstrated that nanostructured lipid carrier-encapsulated formulations achieve a fourfold increase in the intratumoral drug concentration in biliary tract cancer models 113. Although more clinical trials remain necessary for therapeutic validation, tazemetostat represents a pivotal breakthrough in tumor therapy, emphasizing its translational potential to exert broader therapeutic utility in precision oncology.

In conclusion, although third-generation TKIs such as repotrectinib significantly prolong progression-free survival, tumor cells can still restore TRK signaling through secondary mutations in the kinase domain (e.g., G595R, V600E, and L695P). Owing to the sustained tyrosine kinase activity of NTRK fusion proteins, downstream signaling pathways become dysregulated, leading to uncontrolled cell proliferation and genomic instability. Therefore, vigilance against resistance mutations is needed during long-term treatment. The emergence of tazemetostat marked a shift in therapeutic strategy from pure kinase inhibition toward epigenetic modulation and combination therapy. By combining epigenetic regulators such as tazemetostat, drug sensitivity can be enhanced within a specific time window, achieving dual-mechanism blockade of tumor growth and reducing the risk of drug resistance.

### 2.4. Frontier Technology (FT)

#### 2.4.1. Nanomedicine and Nanodelivery

Studies have revealed that compared with their normal counterparts, tumor cells display a significantly heightened metabolic dependence on specific amino acids. This metabolic vulnerability provides a compelling molecular rationale for the development of nanomedicines designed to target amino acid metabolism. Moreover, advanced nanocarrier systems facilitate the precise delivery of therapeutics to tumor sites, thereby maximizing therapeutic efficacy while simultaneously minimizing off-target toxicity in healthy tissues (Fig. 2).

Currently, carrier-free nanodrugs achieve ultrahigh drug loading through the self-assembly of drug molecules, effectively circumventing the toxicity issues associated with traditional carriers. Consequently, they have emerged as a prominent research hotspot, marked by significant breakthroughs in recent years 114. For instance, a nanodrug self-assembled from 5-aminolevulinic acid (ALA), Fe³⁺, and curcumin enables pH-responsive release, facilitating spatiotemporal synergistic photodynamic therapy and chemotherapy 115. This approach significantly enhances drug accumulation while minimizing systemic toxicity. Indeed, recent research has increasingly focused on integrating nanodelivery systems with metabolic interventions. A pivotal 2022 study demonstrated that cell-targeted nanoparticles could be employed to deliver metabolic inhibitors, effectively remodeling the tumor immune microenvironment and augmenting T-cell infiltration. *In vivo* experiments have validated this strategy, revealing a remarkable 75% reduction in pulmonary melanoma metastases 116 (Table 1). Studies have subsequently revealed that ROS-responsive nanocarriers enable the dynamic release of amino acid metabolism modulators. For instance, hyaluronic acid-bilirubin nanoparticles (HA-BR NPs), triggered by ROS, can release methioninase to effectively suppress the progression of BC 117. Recent studies in 2025 have demonstrated that NCT-503@Cu-HMPB has significantly enhanced therapeutic potential. The bioavailability of NCT-503 was substantially enhanced through the utilization of a copper-integrated hollow mesoporous Prussian blue (Cu-HMPB) nanoparticle delivery system. Concurrently, research published in the same year indicates that compared with conventional therapies, water-soluble nanodrug delivery platforms based on amino acid-derived carbon quantum dots offer prolonged systemic circulation, superior tumor-targeting precision, and reduced toxicity 118.

When using traditional chemotherapeutic agents, clinicians often face the dilemma of significant collateral damage to healthy tissues, whereas the core breakthrough of nanotechnology lies in achieving spatiotemporal precision in targeting. Current research has shifted from simple carrier construction to stimuli-responsive designs—utilizing the tumor microenvironment (e.g., pH and ROS levels) to trigger drug release. For instance, targeted delivery of Met inhibitors successfully reduced the lung metastasis of melanoma by 75%; the self-assembled nanodrug ALA/Fe³⁺/curcumin enabled spatiotemporal synergy between photodynamic therapy (PDT) and chemotherapy, and an amino acid-derived carbon quantum dot-based platform significantly prolonged the drug half-life 119-121. Although nanotechnology has demonstrated significant potential in prolonging drug half-life, the complexity of the TME presents substantial physical and physiological barriers. While nanoparticles exhibit prolonged circulation in the bloodstream, their ability to reach tumor sites remains a critical bottleneck. Disorganized vasculature and poor permeability of tumor blood vessels often lead to insufficient perfusion in certain regions. Even when drugs reach the vasculature, high interstitial pressure and a dense extracellular matrix frequently hinder the penetration of nanoparticles into the core tumor tissue. Consequently, drugs may accumulate in the perivascular space rather than entering the interior of tumor cells 122. Although nanomedicines can improve drug delivery efficiency, the complex characteristics of the TME, such as elevated interstitial fluid pressure and a dense extracellular matrix (ECM), still hinder tumor penetration. Furthermore, their long-term biosafety, including *in vivo* metabolic accumulation, remains to be fully validated.

#### 2.4.1. Amino Acid Transporter Inhibitors (AATIs)

Amino acid transporters facilitate the uptake of amino acids to sustain tumor cell growth and proliferation, positioning them as promising therapeutic targets for novel anticancer drug development. Among amino acid transporters, L-type amino acid transporter 1 (LAT1) has been extensively investigated, particularly in pancreatic cancer (PC), where it is markedly overexpressed. Pharmacological targeting of LAT1 effectively suppresses tumor progression 123 (Fig. 2). The LAT1 inhibitor KYT-0353 suppresses the uptake of neutral amino acids, downregulates cyclin D1 expression, and activates the MAPK pathway, thereby inducing G0/G1 phase arrest. Notably, this agent synergistically suppresses pancreatic cancer (PC) cell proliferation when combined with gemcitabine 124. Additionally, KMH-233 induces tumor cell apoptosis while sparing cerebral amino acid homeostasis, demonstrating its ability to penetrate the blood-brain barrier (BBB) in glioma models. Furthermore, its combined use with PD-1 inhibitors enhances antitumor immune responses 123. In addition, JPH203 exerts dual-target inhibition of SLC6A14 (ATB⁰,+) and SLC7A5 (LAT1) transporters, resulting in marked suppression of mTORC1 signaling activity 125. However, LAT1 inhibitors face significant challenges in terms of drug resistance. Prolonged LAT1 inhibition can trigger the activating transcription factor 4 (ATF4) pathway, enabling tumor cells to partially restore amino acid metabolic homeostasis. To date, clinical trials of LAT1 inhibitors have predominantly focused on biliary tract cancers and pancreatic cancer (PC), while other malignancies with similarly high LAT1 expression, such as breast cancer (BC) and gliomas, remain underexplored. This field is still in its nascent stage, with critical hurdles in clinical translation and resistance mechanisms yet to be fully resolved. Given that these agents are currently in early-phase clinical trials, the underlying mechanisms of drug resistance remain insufficiently characterized, and optimal combination therapy regimens, including precise drug dosages and administration timing, have not yet been established. Furthermore, this drug is still in the early phase of clinical trials. Research on the mechanism of drug resistance (e.g., activation of the ATF4 pathway by LAT1 inhibitors) is insufficient, and the optimal regimen for combination therapy (including drug dosage and administration timing) has not yet been clarified.

Amino acid transport inhibitors (AATIs) aim to “starve” cancer cells by blocking the uptake of essential amino acids. The inhibition of transporters such as LAT1 and SGLT1 prevents the cellular entry of neutral and cationic amino acids, thereby inducing G0/G1 cell cycle arrest and suppressing proliferation. However, amino acid deprivation can activate the PERK-eIF2α-ATF4 pathway, leading to the upregulation of downstream genes such as CHOP and ASNS by ATF4. This compensatory response restores amino acid biosynthetic capacity and may even enhance antiapoptotic mechanisms, representing a potential pathway of therapeutic resistance 126. However, amino acid transporter inhibitors exhibit insufficient target specificity, and thus may interfere with amino acid uptake in normal cells, consequently inducing adverse reactions. Moreover, their clinical translation is still in the preliminary stage and lacks support from large-scale clinical trials.

## 3. Discussion and Conclusions

**Figure 4:**
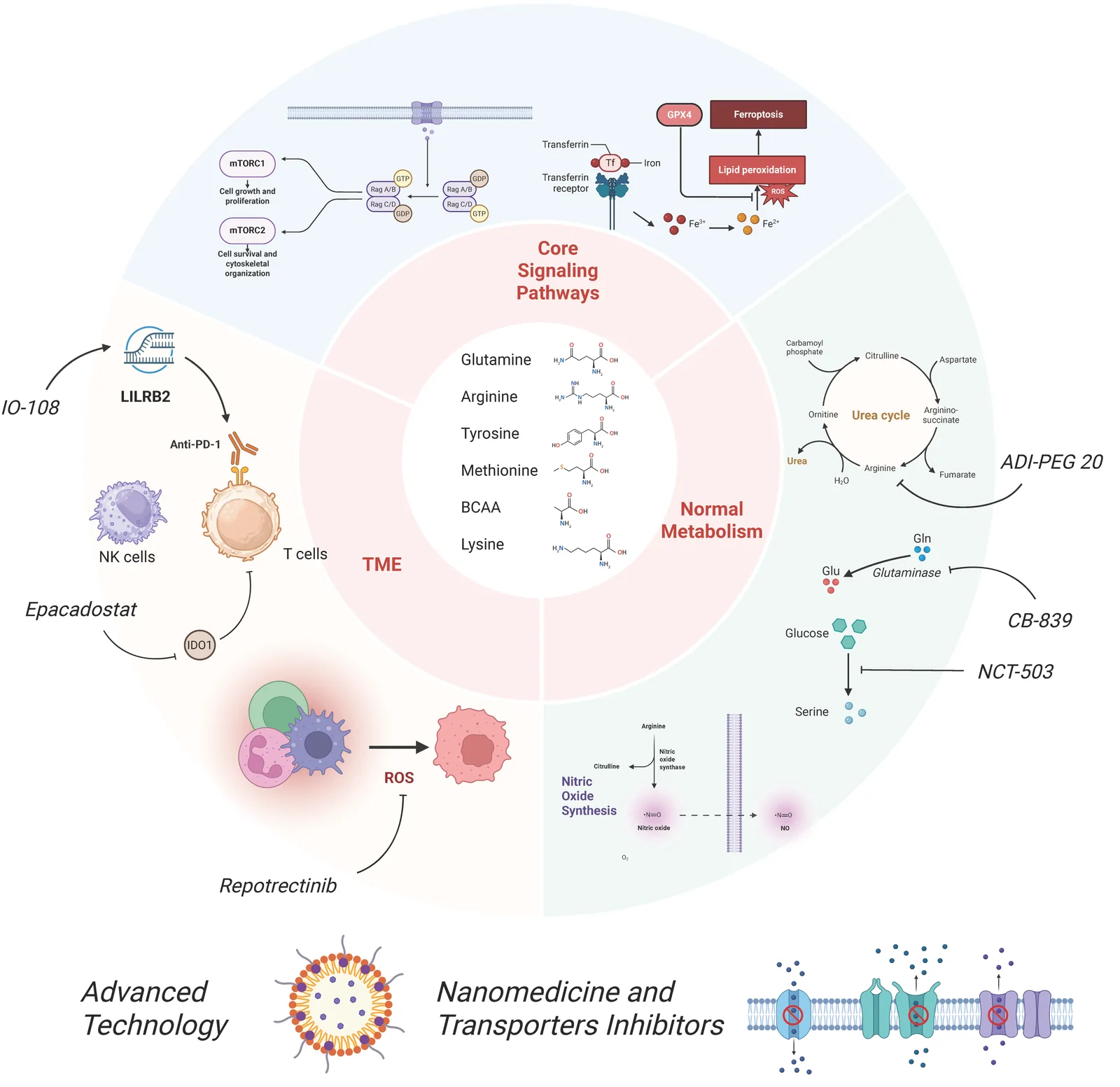
** Overview of the Information Discussed in this Review.** This schematic diagram provides an overview of the entire study, illustrating the key ferroptosis and mTOR signaling pathways, the major immunometabolic and basal metabolic alterations within the tumor microenvironment (TME), and the therapeutic targets of the drugs investigated in this research. The arrows indicate promotion, and the T-bar arrows indicate inhibition.

Metabolic plasticity in the TME is defined as the inherent ability of tumor cells and stromal cells (including cancer-associated fibroblasts and immune cells) to dynamically reprogram their metabolic pathways in response to changes in nutrient availability, oxygen tension, and signaling molecules, thereby maintaining cellular survival, proliferation, and malignant progression (Fig. 4).

In this review, the differential expression of BCKDH across different tumor types (e.g., CRC vs. NSCLC) and the distinct metabolic dependency of various cancers on Met are discussed. However, most existing studies focus on a single tumor type, and systematic comparative research across multiple tumor types and subtypes is lacking. This limitation hinders the validation of the generalizability of amino acid metabolic regulatory mechanisms. Furthermore, the differential roles of the same amino acid in different TMEs—such as the dual NO-mediated effects of Arg in distinct tumors—have not been thoroughly investigated, and the underlying reasons for inconsistent research findings (e.g., variations in sample size and detection methods) remain poorly understood.

In addition, although we have summarized the associations between amino acid metabolism and ferroptosis, mTOR signaling, and immune regulation, most current studies remain correlational and lack rigorous causal validation. For instance, the link between the Met-SAM axis and ferroptosis is mostly limited to phenotypic observations following pharmacological intervention. Met restriction impairs ubiquinone biosynthesis, thereby triggering mitochondrial ROS accumulation and subsequent ferroptosis. Moreover, emerging evidence has demonstrated that MAT2A knockout alleviates chemotherapy-induced cardiomyocyte ferroptosis by restraining SAM production. Mechanistically, SAM-mediated methylation stabilizes GPX4 expression and consequently inhibits ferroptosis-related cell death. Nevertheless, phenotypic validation based on pharmacological interventions (e.g., FIDAS-5 and IKE) or dietary manipulation is currently largely used in fundamental investigations in this field. *In vivo* dynamic monitoring and metabolomic correlation analysis based on clinical samples remain relatively insufficient, which limits the comprehensive understanding of the Met-SAM-ferroptosis regulatory axis 39. Moreover, the regulatory mechanisms underlying the crosstalk among different amino acid metabolic pathways (e.g., the interplay between glutamine (Gln) metabolism and BCAA metabolism) have not been fully elucidated. T cells rely on leucine to activate the mTORC1 signaling cascade and sustain their effector antitumor functions. Tumor cells with elevated LAT1 expression competitively scavenge intratumoral leucine, establishing a metabolically hostile microenvironment that ultimately suppresses T-cell immune competence. Notably, BCKDH expression exhibits context dependent, opposing patterns across distinct tumor subtypes. In colorectal cancer and non-small cell lung cancer, increased BCKDH levels are closely associated with metastatic progression and adverse clinical prognosis. Conversely, in certain preclinical tumor models, BCKDH activity is negatively modulated via BCKDK-mediated phosphorylation, which facilitates BCAA accumulation and sustains tumor metabolic reprogramming. From a therapeutic perspective, glutamine-targeting agents such as CB-839 have yielded promising antitumor efficacy in specific cancer contexts, whereas pharmacological interventions targeting BCAA metabolism exert a context-dependent dual, double-edged effect on tumor regulation 127. These discrepancies remain to be further studied in future investigations.

Amino acids beyond those extensively studied also fulfill essential functions within the TME. Serine and glyci**n**e, in addition to their roles as one-carbon donors in redox regulation and epigenetic modulation, are subject to circadian control according to a 2025 study. The BMAL1/Clock complex was found to regulate the diurnal expression of serine/glycine synthesis and transport genes (e.g., PHGDH, PSAT1, and SHMT). During phases of peak BMAL1 expression (morning/afternoon), increased serine supply promotes tumor proliferation and affects drug sensitivity 128. It is anticipated that SHMT inhibitors, such as SHIN1, not only inhibit proliferation in multiple solid tumors (e.g., breast, pancreatic, and colorectal) but also sensitize them to chemotherapy and/or immunotherapy, thereby improving treatment outcomes 129. However, the field is currently hindered by several major challenges, including tissue-specific heterogeneity, unclear causal relationships, and insufficient temporal resolution. A newly proposed mechanism of metabolism‒translation coupling suggests that in brain tumors, particularly GBM, threonine metabolism fuels tumor proliferation through YRDC-mediated t⁶A tRNA modification, thereby enabling the preferential translation of codon-biased transcripts. Promising intervention strategies—including dietary restriction, YRDC inhibition, SLC1A5 blockade, and their combination with immune checkpoint blockade—offer multilayered, metabolically targeted approaches for clinical translation 130. Interventions targeting these mechanisms are emerging as key strategies for preventing immune suppression and restoring immune surveillance.

The dual characteristics of targeted amino acid metabolic therapy remain poorly defined. Although previous reviews have highlighted the advantages of such therapeutic strategies, their side effects are also evident. CB-839 can target and inhibit GLS1 to suppress tumor growth; however, long-term administration may impair glutathione (GSH) synthesis in normal cells, thereby inducing oxidative stress damage. ADI-PEG 20 can be used only for ASS1-deficient tumors, resulting in a narrow patient population, and drug resistance to ADI-PEG 20 is also common. The side effects associated with these agents warrant further in-depth investigation and resolution in future studies.

Finally, it is necessary to strengthen research on metabolic heterogeneity across different tumor types and subtypes and construct multicenter, large-sample databases of amino acid metabolism data. These efforts will help clarify the core regulatory mechanisms of amino acid metabolic pathways in distinct tumors and resolve the inconsistencies among existing research conclusions. In addition, an in-depth exploration of the regulatory network among amino acid metabolic pathways is needed to elucidate the synergistic mechanisms underlying metabolism-immune-epigenetic regulation, thereby overcoming the limitations of single-pathway research. Furthermore, clinical study designs should be optimized to further investigate drug resistance mechanisms and explore personalized combination therapeutic strategies (e.g., metabolic inhibitors combined with immune checkpoint inhibitors and nanodelivery systems) to improve the efficiency of clinical translation. Moreover, the development of high-sensitivity and high-throughput metabolic detection technologies and the identification of biomarkers capable of precisely evaluating the metabolic status of the TME are urgently needed to provide solid support for precise clinical tumor therapy.

## Figures and Tables

**Figure 3:**
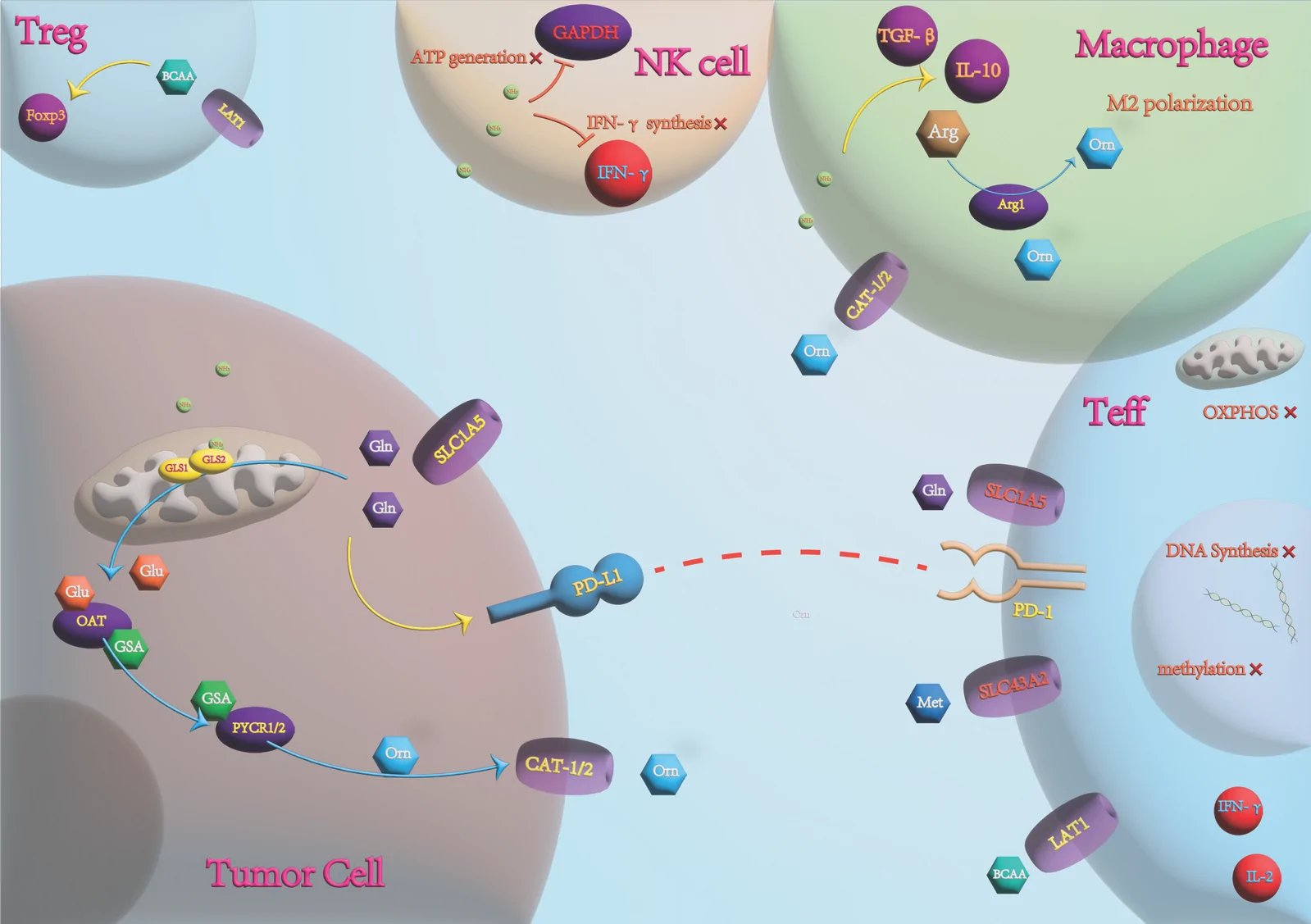
** Overview of the Immune Microenvironment.** The blue arrows indicate direct transformation, the yellow arrows represent indirect activation, and the T-bar arrows denote inhibition. IFN-γ, Interferon-gamma; TGF-β, Transforming growth factor-beta; CAT, Cationic amino acid transporter; OXPHOS, Oxidative phosphorylation; LAT1, L-type amino acid transporter 1; PYCR, Pyrroline-5-carboxylate reductase.

**Table 1:** Summary of anticancer agents targeting amino acid metabolism and related pathways.

	Drug	Targeted AA	Targeted Pathway	Targeted Points	Clinical Trial Stage	Combination Therapies	Cancer	Challenges/Discoveries
AMT	CB-839	Gln	TCA	GLS1	II (NCT03428217)	Cabozantinib	RCC	Adverse events and absence of a significant monotherapy efficacy signal
					I/II (NCT03263429)	Panitumumab	CRC	
					II (NCT03263429)	5-FU	CRC	
					Preclinical	Metformin	OS	
	ADI-PEG 20	Arg	Urea Cycle	ASS1	I (NCT03254732)	Pembrolizumab	Solid	Toxicity to neutrophils
	NCT-503	Ser	SGOC	PHGDH	Preclinical	PKM2 inhibitor	NSCLC	Dual inhibition of PKM2 and PHGDH
IO	Epacadostat	Trp	IDO1-KYN	IDO1	III (KEYNOTE-434)	Pembrolizumab	Solid	High risk of toxicity
	IO-108	Trp	LILRB2-MHC	LILRB2	I (NCT05054348)	Pembrolizumab	Solid	Safety but moderate efficacy
	EPZ015666	Arg	PRMT5-SDMA	PRMT5	Preclinical	As_2_O_3_	APL	Methylation of the E3 ligase RNF4
TT	Repotrectinib	Tyr	ROS1-NTRK-	ROS1	III (KEYNOTE-434)	No Data	NB	High risk of toxicity
	Tazemetostat	Lys	PRC2-	EZH2	I/II (NCT04624113	Pembrolizumab	HNSCC	Lack of synergistic effect and extremely low ORR
			H3K27me3		I (NCT04917042)	Topotecan	SCLC	
FT	Nanomedicine	Met	Met-SAMe	MAT2A	Preclinical	***No Data***	Melanoma	Targeted drug delivery
	AATI	Amino	AATs	LAT1	***No Data***	***No Data***	PC/LC	Tumor-Specific starvation

No Data: There is currently no available data supporting. AMT: Amino acid metabolism-targeted drugs; IO: Immune-oncology agents; TT: Targeted tyrosine kinase/ epigenetic therapies; FT: Frontier Technology. Drug: Compound name. Targeted Amino Acid: Specific amino acid metabolism pathway disrupted. Targeted Pathway: Key metabolic/ signaling pathway affected. Targeted Points: Molecular targets. Clinical Trial Stage: Current development phase. Combination Therapies: drug combinations. Cancer: Tumor types under clinical evaluation. SGOC: Serine-glycine-one-carbon metabolism; IDO1-KYN: indoleamine 2,3-dioxygenase 1-kynurenine axis; PRMT5-SDMA: protein arginine methyltransferase 5-symmetric dimethylarginine; ROS1-NTRK-ALK: receptor tyrosine kinase fusions; PRC2-H3K27me3: polycomb repressive complex 2-histone H3 lysine 27 trimethylation; ISR: integrated stress response.
